# The coverage and frequency of mass drug administration required to eliminate persistent transmission of soil-transmitted helminths

**DOI:** 10.1098/rstb.2013.0435

**Published:** 2014-06-19

**Authors:** Roy Anderson, James Truscott, T. Deirdre Hollingsworth

**Affiliations:** 1 London Centre for Neglected Tropical Disease Research, Department of Infectious Disease Epidemiology, School of Public Health, Faculty of Medicine, Imperial College London, St Marys Campus, Norfolk Place, London W2 1PG, UK; 2 Mathematics Institute, University of Warwick, Coventry CV4 7AL, UK; 3 School of Life Sciences, University of Warwick, Coventry CV4 7AL, UK; 4 Department of Clinical Sciences, Liverpool School of Tropical Medicine, Pembroke Place, Liverpool L3 5QA, UK

**Keywords:** soil-transmitted helminths, modelling, elimination, chemotherapy, school-based intervention

## Abstract

A combination of methods, including mathematical model construction, demographic plus epidemiological data analysis and parameter estimation, are used to examine whether mass drug administration (MDA) alone can eliminate the transmission of soil-transmitted helminths (STHs). Numerical analyses suggest that in all but low transmission settings (as defined by the magnitude of the basic reproductive number, *R*_0_), the treatment of pre-school-aged children (pre-SAC) and school-aged children (SAC) is unlikely to drive transmission to a level where the parasites cannot persist. High levels of coverage (defined as the fraction of an age group effectively treated) are required in pre-SAC, SAC and adults, if MDA is to drive the parasite below the breakpoint under which transmission is eliminated. Long-term solutions to controlling helminth infections lie in concomitantly improving the quality of the water supply, sanitation and hygiene (WASH). MDA, however, is a very cost-effective tool in long-term control given that most drugs are donated free by the pharmaceutical industry for poor regions of the world. WASH interventions, by lowering the basic reproductive number, can facilitate the ability of MDA to interrupt transmission.

## 1. Introduction

**Figure 1.**
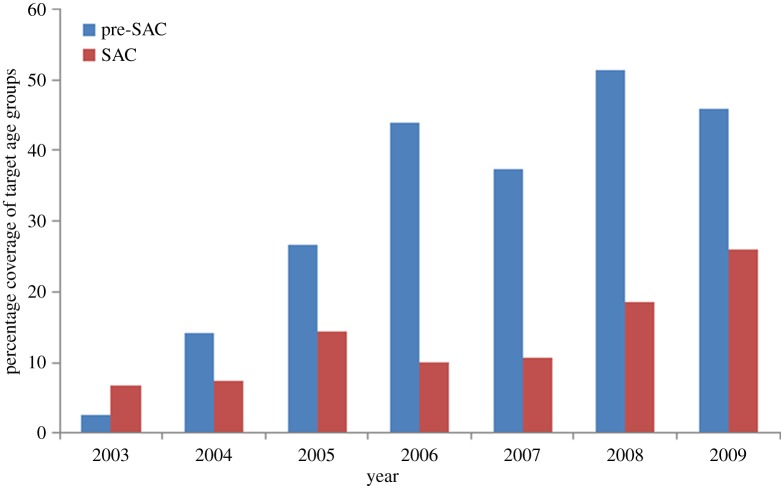
Coverage of preventive STH chemotherapy in pre-school-aged children (pre-SAC) and school-aged children (SAC). WHO African Region, by year, 2003–2009 [1]. (Online version in colour.)

Funding for the control of soil-transmitted helminths (STHs) by mass or targeted chemotherapy in developing countries has increased steadily in the past 10 years (figure 1). This is due to generous donations from international aid agencies in the richer countries, philanthropic organizations and pharmaceutical companies. The spirit of this expanded effort is captured in the London Declaration in January 2012, and the progress reported 1 year later [2,3]. The effort is part of a broader push to bring a range of neglected tropical diseases (NTDs) under control, and in some cases to aim for elimination [4].

Many questions remain about how best to deliver community-based treatment programmes for the various treatable NTDs to obtain the greatest impact from recent drug donations to the World Health Organization (WHO) and specific countries. These include the following: who should be treated, how often should they be treated, can treatment intervals be increased as worm loads fall, and can transmission be eliminated by repeated treatment alone [5]? To answer these questions, a detailed understanding of the transmission dynamics of the parasites is required, because community-based treatment acts as a perturbation to the parasite's population dynamics and stability.

WHO guidelines for the community-based treatment of STHs are based on the consensus views of experts. WHO has chosen prevalence as the main measure for programme design at initiation of chemotherapy, and for monitoring impact in the community. As discussed in §2, this is not the ideal measure owing to a highly nonlinear relationship between prevalence and the mean worm load created by the aggregated distributions of worm numbers per person [5–8]. WHO does suggest intensity measures where possible, and this may become the preferred outcome measure for monitoring impact in the near future.

Four drugs are recommended by WHO for treatment: albendazole (dose of 400 mg) and mebendazole (dose of 500 mg), which are most widely used in programmes, levamisole (dose of 2.5 mg kg^−1^) and pyrantel (dose of 10 mg kg^−1^), which are less commonly used. All are well-known, safe and effective drugs that have been used widely in recent years for the treatment of *Ascaris lumbricoides*, *Trichuris trichiura* and hookworm (*Ancylostoma duodenale* and *Necator americanus*). A single dose oral anthelminthic treatment can also be given to pregnant and lactating women, but, as a general rule, drugs should not be given in the first trimester of pregnancy. In practice, however, few countries have included anthelmintics in their antenatal care programmes, with only Nepal and Sri Lanka doing so routinely.

Three strategies are suggested for the use of chemotherapy in the treatment of infections of STH in the community. The first is universal population-level application of anthelminthic drug, in which the community is treated irrespective of age, sex, infection status or other social characteristic. The second is targeted group-level application of anthelminthic drug where the group may be defined by age, attendance at school or other social characteristic, typically occupation, irrespective of infection status. The third is selective individual-level application of anthelminthic drug where selection is based on diagnosis of current infection (see [9,10] for early analyses of the relative impact of these approaches). This is not considered in our analyses because of the many practical issues associated with its delivery.

Six questions are examined in detail in this paper through data analysis, mathematical model development, parameter estimation and numerical analysis. A key aim is to evaluate current WHO guidelines on STH control by anthelmintic drug administration. The questions are as follows.

Can community-based chemotherapy alone stop transmission of STHs within defined populations? How frequently should treatment be administered? What level of treatment coverage is required to lower average worm burdens to low levels? Can the intervals between treatments be lengthened once worm burdens fall to low levels? Can transmission be halted by treating school-aged children (SAC) alone, or by treating both SAC and pre-SAC? What are the most appropriate measures of control impact? All questions are assessed for different transmission settings (as measured by *R*_0_—low, medium and high), different values of key parameters (severity of density dependence, *z*, different age group contributions and exposure to infective stages in the habitat, the degree of parasite aggregation, *k*) and for different species mixes of STHs in a given location. We address these questions by both reviewing existing work and presenting novel analyses.

## 2. Methods

### (a) Outcome measures

A variety of outcome variables can be measured via cross-sectional (stratified by age) or longitudinal epidemiological studies. The most commonly recorded is the prevalence of infection, but for the reasons outlined below, this is of limited value. The best measure is the intensity of infection measured either directly by counting worms expelled in the faeces of treated persons, or indirectly by eggs per gram measures (epgs). The latter is most frequently used owing to the workload involved in collecting faeces and counting worms by sieving the faecal material. For hookworm and *Trichuris*, such counts are subject to error owing to the small size of the expelled worms. Counts of eggs by Kato Katz or McMaster methods are also subjected to much variability in observation [11], and repeated counts on the same faecal output or pooling methods are required to attempt to minimize these [12]. The distribution of epg in repeated counts from the same faecal output from one patient follows a negative binomial distribution. Compounding this variability across counts from different patients, even when in the same age class, results in greater degrees of aggregation with very low *k*-values.

**Figure 2.**
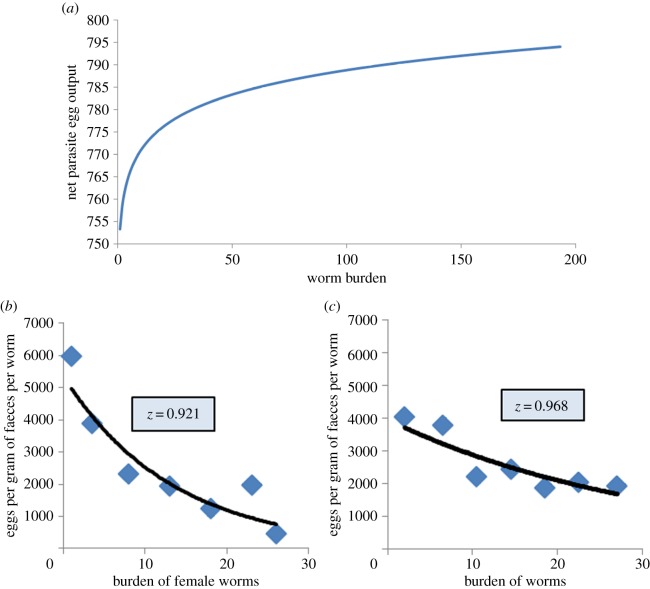
(*a*) Relationship between total egg output per host and worm burden for hookworm predicted by observed density-dependent relationships between egg output and worm load per patient (*z* = 0.95, where *z* = exp(−*γ*)). (*b*) Density-dependent fecundity for *Ascaris* from the studies of Holland *et al.* [13] in Nigeria and (*c*) Elkins *et al.* [14] in India with *z*-values inset. (Online version in colour.)

A further complication arises from the density-dependent nature of egg production by helminths, where per female worm egg output falls (often exponentially [7]) as worm load in the host rises. Figure 2 illustrates this effect by reference to total egg output per host as a function of worm load for hookworm. An obvious consequence of this effect is that reductions in mean worm load have a reduced effect on egg intensity measures, unless the mean load is close to the breakpoint in transmission (where *R*_0_ is close to unity).

In assessments of control impact, a number of different outcome measures can be used. These are the epidemiological statistics of prevalence and mean intensity of infection, and a number of other measures such as the force or rate of infection per host (see §2*b* for definition), the basic reproductive number that measures transmission success in the absence of control, *R*_0_ and the effective reproductive number that measures transmission success under the impact of control, *R*_e_. In this study, we use temporal changes in average worm loads over the period of the control programme and post its cessation to assess impact of a defined control programme as predicted by mathematical models.

We also include our simulated treatment programmes in the context of WHO guidelines on low, medium and heavy infection categories based on epg counts, using published data on egg production per worm.

Given the observed aggregated distributions of worm numbers per person [7,8], which are well described by the negative binomial frequency distribution, the relationship between prevalence (as a proportion infected), *p*, and the mean worm burden, *M*, is given by
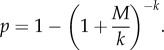
Here, *k* denotes the aggregation parameter of the negative binomial, which inversely measures the degree of clumping. Typical values for intestinal helminths lie in the range 0.1–0.9, which reflects a high degree of aggregation. One consequence of this is that changes in the mean intensity by many factors (four or more), perhaps owing to community-based chemotherapy programmes, have little impact on the prevalence of infection. This feature leads to the conclusion that prevalence is a very poor outcome statistic by which to measure the impact of control programmes [8].

**Figure 3.**
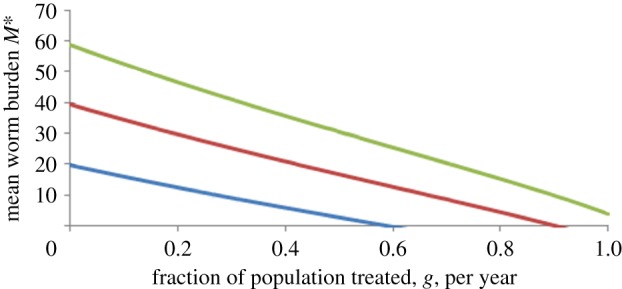
Relationship between the proportion of the population treated per year, *g*, and the post-treatment equilibrium worm load (for, from bottom to top, *R*_0_ = 1.5, 2.0 and 2.5). (Online version in colour.)

The approximate relationship between the fraction of a population treated per unit of time, *g*, with a drug of efficacy, *h*, and mean intensity of infection, M*, at the new equilibrium created by the repeated treatment after many rounds of chemotherapy is given by [6]2.1
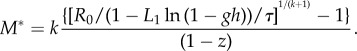
Here, *z* = exp(−*γ*), where *γ* denotes the severity of decay in egg output per worm as mean load rises (figure 2), *τ* is the interval separating treatments and *L*_1_ is adult parasite life expectancy. A plot of this function (M* versus *g*) is presented in figure 3, with parameter values to mimic hookworm infection, with drug efficacy set at 0.8 (80%), density dependence as defined in figure 2 with *R*_0_ varying from 1.5 to 2.5.

Note that despite the apparent complexity of equation (2.1), the relationship between M* and *g* is roughly linear. Also note that for low *R*_0_ values (e.g. 1.5), less than 100% of the community need treating repeatedly to eradicate infection (eliminate transmission where *R*_0_ > 1).

### (b) Force of infection

The *per capita* force of infection, *Λ*, is defined as the rate at which humans acquire mature worms, taking into account survival from infection to maturity (delay *τ*_2_) in the human host when the worm starts to produce eggs—believed to be around 50–80 days for *Ascaris* and 28–50 days for hookworm. It is an important measure of the transmission potential of the parasite prior to and post-treatment, and can be measured in a variety of ways. Typically, it is dependent on host age [15], but for the purpose of simplicity, we assume an average rate over all age classes. In these circumstances2.2
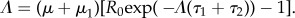
Here, *R*_0_ is the basic reproductive number, 1/*μ* human life expectancy, 1/*μ*_1_ adult parasite life expectancy, *τ*_1_ the average time from egg release to maturation to the infective state and *τ*_2_ is the maturation delay of the parasite in the human host.

Where the maturation delays (*τ*_1_ and *τ*_2_) are short with respect to adult worm life expectancy (1/*μ*_1_), this simplifies to2.3
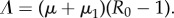


If worm life expectancy (*L*_1_ = 1/*μ*_1_) is short (a year or two) in relation to human life expectancy (many decades), equation (2.3) simplifies to2.4
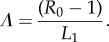


The per host time unit of equation (2.4) is set by the unit of time set to measure worm life expectancy.

For a study of *Ascaris* in a rural community in Taiwan [16], Anderson [15] estimated the *per capita* per annum infection rate, *Λ*, to lie between 0.5 and 9 depending on host age (large values for older age groups). From a study of *Ascaris* in Iran where transmission was intense, Croll *et al.* [17] gave an estimate of the per annum infection rate of 22 worms per host.

The magnitude of the force of infection (hence the magnitude of *R*_0_) sets the time over which worm loads will bounce back to pre-control levels after a round of chemotherapy. The higher the value of *Λ*, the faster is the bounce back time. The force of infection therefore determines the optimal intervals between rounds of treatment. For a simple deterministic model, equation (2.4) makes clear how this rate of reinfection depends simply on the basic reproductive number, *R*_0_, and parasite life expectancy, *L*_1_. The higher the value of *R*_0_, the more frequent must be treatment rounds to sustain low average worm burdens. Long adult worm life expectancies imply less frequent rounds of treatment for a fixed *R*_0_. This is because adult worm life expectancy acts inversely to determine the rapidity of turnover of the adult parasite population, and as such dictates how often treatment needs to be administered.

More complex models that represent mating probabilities and age class-dependent exposure reveal more complex relationships for bounce back times, with the rate of reinfection being low when worm loads are reduced near to a ‘breakpoint’ in transmission. Numerical studies are required to delineate this trend, and fuller details are presented in a future publication.

### (c) Data sources

At present, our knowledge of the key parameters controlling the transmission dynamics (and hence response to community-based chemotherapy) of STHs is limited, with many of the published estimates deriving from studies in the 1980s or earlier [7].

Table 1 presents estimates of the four major epidemiological parameters, namely parasite life expectancy, density dependence in fecundity, parasite aggregation (negative binomial parameter, *k*) and the basic reproductive number, *R*_0_. In the numerical studies of mathematical model behaviour under various assumptions concerning treatment intensity and frequency, we use the estimates listed in table 1, unless otherwise stated.

**Table 1.** Estimates of the key epidemiological parameter*s*.

parasite	*R*_0_	*k*	density dependence, *z* [=exp(−*λ*)]	adult worm life expectancy (years)	region	reference
*Ascaris*		0.81	0.968	1	India	[14]
*Ascaris*			0.927		Nigeria	[13]
*Ascaris*	4–5	0.57	0.991		Iran	[17]
*Ascaris*		0.6–0.7			Bangladesh	[18]
*Ascaris*	1–3	0.46			Myanmar	[19]
*Ascaris*		0.59			St Lucia	[20]
*Ascaris*	1–2	0.44			Bangladesh	[21]
*Ascaris*		0.36–0.54			South Korea	[22]
*Ascaris*		0.54			many countries	[23]
*Ascaris*		—	0.992		Malaysia	[24]
*Ascaris*		0.2–0.5			Japan	
hookworm		0.45			Papua New Guinea	[25]
hookworm					India	[26,27]
*Ancylostoma*		—		1		
*Necator*	2–3	0.03–0.6		3–4		
						
*Necator*		0.16–0.24			India	[7,28]
*Necator*		0.05–0.4			Taiwan	[15]
*Necator*	3–4				China	[29]
*Necator*	2	0.35	0.92		Zimbabwe	[30]
*Trichuris*	8–10	0.2–0.4			St Lucia	[20]
*Trichuris*	4–6				Jamaica	[31]

**Figure 4.**
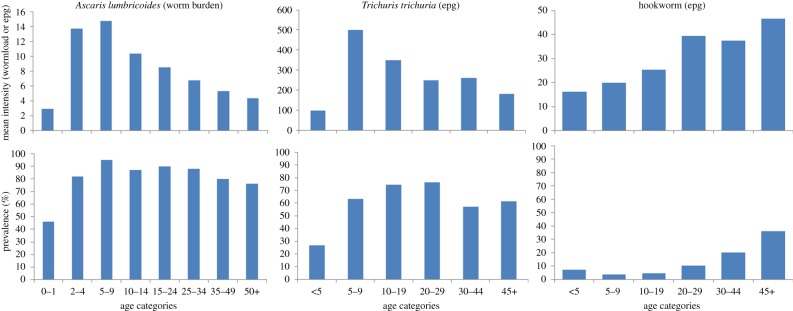
Age–intensity profiles for mean intensity and prevalence (%) for *Ascaris* [19], *Trichuris* [33] and hookworm [34]. (Online version in colour.)

The expanded efforts to control STHs currently underway present opportunities to add to our knowledge of these key parameters. For example, monitoring reinfection (by intensity measures, not prevalence), as illustrated by the studies of *Ascaris* by Hliang *et al.* [32] and Elkins *et al.* [14], can provide information on which to estimate the force of infection, *Λ*. Observed patterns of change of prevalence and intensity with age vary greatly by species of STH and by study location. However, some general trends are apparent from a wide range of published surveys from many different countries as illustrated in figure 4. *Ascaris* tends to rise to peak intensity in 5–14 year olds and then decline in adults. By contrast, hookworm intensity tends to continue to rise through adult life, where most parasites are harboured in the adult age classes. *Trichuris* is somewhat intermediary between the two—but perhaps more towards the convex curves for *Ascaris* where intensity falls in adult age classes. For all three species, prevalence rises rapidly and approaches a plateau in SAC to remain at a plateau in adult life. This contrast between the two epidemiological statistics (two parameters of the frequency distribution of parasite numbers per host) highlights why intensity is a much better measure than prevalence as a reflection of burden, transmission activity and the impact of control measures throughout the age groups.

The reasons for convexity in age–intensity profiles remain poorly understood. The question of whether it is due to ‘ecology (age-dependent exposure) or immunology (acquired immunity)’ remains unanswered [35]. The truth is likely to be a combination of both processes. For each STH species, these patterns of change with age appear to change very little with respect to the overall intensity of transmission in a given habitat. This observation may suggest that acquired immunity is less important than age-dependent exposure.

Age-dependent exposure may result from a variety of factors, but for the STHs, movement behaviour and defecation behaviour with respect to the spatial distribution of infective stages in a habitat, is clearly very important. The mathematical model defined below takes explicit account of such behavioural and spatial factors by the three major age groups of individuals (pre-SAC, SAC and adults). Statistical fitting procedures (Monte Carlo Markov chain (MCMC) methods) are used to estimate these age-dependent rates of exposure to infective stages for the three age groupings from age–intensity profiles. MCMC methods are used to fit the model to age-related patterns of infection, to estimate both transmission intensity (measured by the basic reproductive number, *R*_0_, the average number of offspring produced by one female worm that survive to reproductive maturity) and age-related exposure to the common infective stage pool.

### (d) Demography

Demographic data are obtained from the US Census Bureau to calculate the fraction of total country populations in the infant class (0–1), pre-SAC class (2–4 years), SAC (5–14 years) and adults (15+ years) [36]. A representative summary of this information is presented in table 2, and these figures are used in our analyses of different treatment programmes targeted at some combination of these three age groups. For countries with endemic STH infection, typical figures are 10–15% in pre-SAC, 20–30% in SAC and 55–75% in adult age groups. Depending on the age profiles for the intensity of infection (figure 4), these figures suggest that less than 50% of worms will be exposed to treatment if chemotherapy is only targeted at the pre-SAC and SAC [8].

**Table 2.** Percentages of total population in pre-school-aged classes, school-aged classes and all >15 years of age in 2013*.*

country	0–1 (infants)	2–4 (pre-school)	5–14 (school age)	15+ (adults)
Bangladesh	4.2	6.4	22.4	67.0
Botswana	4.4	6.6	22.2	66.9
Ethiopia	7.1	9.8	27.5	55.7
India	3.9	5.8	19.2	71.2
Iran	3.5	5.0	15.2	76.3
Jamaica	3.7	5.6	19.7	71.1
Kenya	6.0	9.3	27.1	57.7
Myanmar	3.6	5.3	17.8	73.3
St Lucia	2.8	4.2	14.6	78.4
Uganda	8.2	11.0	29.7	51.1
UK	2.4	3.6	11.2	82.7
Zimbabwe	6.0	8.0	25.4	60.6

### (e) WHO guidelines

The current guidelines for community-based treatment for STH infections based on repeated rounds of chemotherapy are detailed in two reports published in 2002 and in 2011 [37,38].

In brief, these documents define low–medium and high transmission locations as ones in which the prevalence of infection with any STH is less than 50% and at least 50%. If intensity measures based on epgs of faeces are performed, then the classification suggested is detailed in table 3 of light, medium and heavy epg counts. Average epgs may be used either across multiple samples per stool or multiple samples from stools collected on different days from the same patient.

**Table 3.** Classification of intensity of infection for individuals by STH species based on WHO guidelines.

parasite	light epg	medium epg	heavy epg
*Trichuris trichuria*	1–999	1000–9999	≥10 000
*Ascaris lumbricoides*	1–4999	5000–49 999	≥50 000
hookworm	1–1999	2000–3999	≥4000

**Figure 5.**
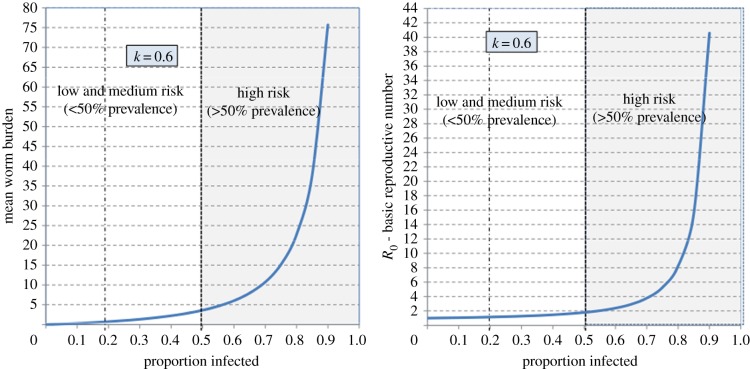
Approximate relationships for soil-transmitted helminths between prevalence (as a proportion) and the mean worm burden, and prevalence and the basic reproductive number *R*_0_ (simple relationship—no mating function, no age structure, see Anderson & May [7]). (Online version in colour.)

These classifications are designed to guide country-based programmes on the frequency of treatment required in given settings. Current practice is the treatment of SAC twice a year where prevalence is more than 50% and once a year where prevalence is 20–49%. Many alternatives exist such as (i) treatment of SAC and pre-SAC once or twice a year; (ii) treatment of an entire community once or twice a year; (iii) treatment of SAC at more frequent intervals than once/twice a year and (iv) treatment of SAC at less frequent intervals than once/twice a year. We examine these alternatives in §3. However, from a methodological standpoint, careful examination of the prevalence classification raises a number of issues of great importance to the design of effective treatment and monitoring programmes. These are illustrated in figure 5, which displays the relationship between the prevalence of infection and two epidemiological parameters, the mean intensity of infection and the basic reproductive number, *R*_0_ (both are direct measures of the intensity of transmission). The relationship shown is based on a negative binomial distribution of parasites per host with an aggregation parameter, *k*, assigned a value of 0.6 which is typical for many STHs (table 1).

These relationships are highly nonlinear and imply that the low and medium prevalence band (less than 50% prevalence) reflects low transmission intensity areas (not low and medium). The high prevalence band (more than 50% prevalence) reflects both medium, high and very high transmission intensity areas, and as such a finer stratification of this class is suggested if the right frequency of treatment is to be calculated to reduce burdens to very low levels or below the transmission breakpoint (created by the mating frequency issue for dioecious parasites; see [7]). If the parasites are more highly aggregated (*k*-values around 0.2–0.1; table 1), as is often the case for hookworm and *Trichuris* infections, this problem becomes more acute, with more than 50% prevalence covering low, medium and high transmission intensity communities. Some revision of the guidelines is suggested by figure 5, with intensity measures replacing prevalence to define low, medium and high transmission intensity communities.

### (f) Mathematical model

We use a deterministic model to represent the dynamics of worm burden in four contiguous age classes: infants (0–1 years of age), pre-SAC (2–4 years), SAC (5–14 years of age) and adults (all more than 15 years old). The 0–4 age range is split into two, because only the pre-school categories (2–4) are eligible for treatment. Previous work [39] has analysed the dynamics of two age class models (less than and greater than 15 years) under regular treatment. However, in the present case, the short age ranges that are a feature of this model are comparable to worm lifespans (1–2.5 years). Hence, we use an explicitly age-structured model and superimpose our desired age structure on it.

The fundamental model used to describe the evolution of the worm burden of individuals of age *a* is taken from Anderson & May [6].2.5
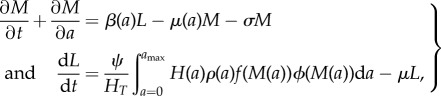
where *M*(*a*) is the mean worm burden in age group *a* and *L* is the volume of infectious material in the environment to which individuals are exposed. The function *f*(.) captures the density dependence of fecundity, and *ϕ* is a reduction factor accounting for the effects of sexual reproduction of worms in the host [6].
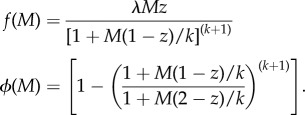


Here, *k* is the shape parameter of the assumed negative binomial distribution of worms among hosts (varying inversely with the degree of clumping) and *z* is the density-dependent fecundity parameter as above (it is assumed that the model is in terms of female worms and that the effect of fecundity is dependent on the host burden of *female* worms).

The effects of host behaviour are encapsulated in the age-dependent parameters *ρ*(*a*), which govern what fraction of an individual's egg output enters the reservoir, and the *β*(*a*), which govern the degree of exposure of the various age groups to the reservoir. Only the relative values of these parameter vectors are important. The absolute size of overall exposure is just one element of the parameter grouping that defines *R*_0_ and hence is a factor of the *R*_0_ value chosen. In the simulations used in this paper, we have used a demographic profile to match the population of Uganda [34]. The demography of the host population is described by the survival function, *H*(*a*), representing the probability for an individual to reach age *a*. The survival function is related to the mortality, *μ*(*a*), through
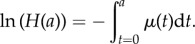


The parameter *H_T_* = ∫*H*(*a*)d*a*. For this model, the value of *R*_0_ is given by the expression
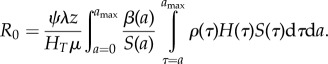
The parameter *S*(*a*) is the survival function for a worm recruited into a host at birth.
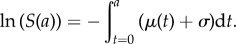


Owing to stratification of the data, we use a discretized version of the evolution equations with separate equations for the worm burden in annual age classes. The model has the form2.6
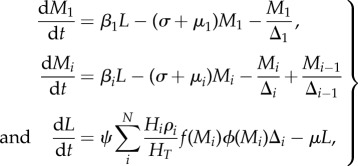
where 1 ≤ *i* ≤ *N*. The parameter ▵*_i_* is the width of the *i*th age class. We use annual age classes, so ▵*_i_* = 1 and *N* = 70. Age-dependent parameters, such as *β* and *ρ*, are discretized into *N*-values, one for each age class. Similarly, the expression for *R*_0_ is approximated by summations in the place of integrals. Age-dependent parameters have distinct values within each of the broad age classes described above (infant, pre-SAC, SAC, adult). For example, *β*_2_, *β*_3_ and *β*_4_ all have the *β*-value assigned to the pre-SAC age group. Because only the relative values of *β* and *ρ* are important, we arbitrarily define *β*- and *ρ*-values to be unity for the SAC group.

Treatment efficacy in an age group is the product of the fraction of the age group enrolled in treatment, and the mean fraction of worms eliminated from treated hosts. Treatment efficacy is treated in the same manner as *β* and *ρ*, with distinct levels of treatment in each of the four age categories giving an efficacy, *γ_i_*, in the *i*th annual age group (treatment efficacy for infants is zero, by definition). Treatment is applied at regular intervals and reduces the mean worm burden in a class by a factor *γ_i_*. To ascertain whether a particular treatment age profile and interval resulted in eradication of the parasite, the model was run from its treatment-free equilibrium through a sequence of treatment intervals lasting 25 years. For a given level of treatment in the pre-SAC and adult age groups, a bisection algorithm was used to identify the lowest level in treatment for the SAC group that resulted in long-term eradication.

### (g) Parameter estimates

The majority of parameter values for the model described in §2*f* were taken from within the ranges found in the literature. Parameter estimates are quite sparse owing to the difficulties of measurement. Table 1 gives a brief survey of values for *k*, *z* and *R*_0_ across different studies and species. While variability is wide, there are clear differences between species. However, data for the age-specific contact rate of hosts with the infectious reservoir (*β*) and age-specific contribution of hosts to the reservoir (*ρ*) are unknown. These were estimated by fitting the model to worm burden age profile data [14,30]. The age-dependent variable, *M*, in our model represents the mean of a negative binomial distribution, making it straightforward to construct a likelihood for a given set of data. In each case, other parameters were chosen to match species natural history and the survival profile of the host population in the area of the study and at the time it was carried out. Using MCMC methods, we identified the maximum-likelihood estimators for *R*_0_ and *β* in the three observed age categories. The MCMC chain was constructed using the MCMC package in R (v. 2.15.1). The values of *ρ* have no effect on the shape of the endemic worm burden age profile, but do have an effect on the transmission dynamics following treatment and reinfection. We therefore investigate two scenarios, first that the rate of contact with the infectious reservoir is proportional to the contribution to the reservoir of a given age class: hence 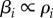 for observed age class, *i*, following the analysis first presented by Truscott *et al.* [40]; second, as a contrast, the assumption that the deposit rate into a shared pool is the same for *ρ* = 1 for all groups, we further investigate not only a fixed programme duration, but also variable programme duration.

## 3. Results

We present the critical treatment coverage as a three-dimensional surface of the effective treatment combinations of pre-SAC, SAC and adults that results in crossing the critical treatment surface to extinguish parasite transmission (values equal to or above the surface cross the ‘breakpoint’ (an unstable equilibrium) and lead to long-term extinction in the absence of immigration). Results in the format of look-up tables of the values within these plots may be more useful to public health workers [40]. As described below, the surfaces do not give information on the temporal dynamics of the infection, and we therefore investigate these using both time series of repeated treatment of different proportions of the three treatment age groupings and summaries of the number of rounds of treatments required to reach the breakpoint for different coverages.

The proportion effectively treated that is needed to reduce mean worm burdens below the critical level are calculated for *R*_0_ set at low (2), medium (3) and high (5) values, and for different parasites. The parasite with the greatest *R*_0_ value determines the intensity, frequency and duration of treatment required. Graphs are for *Ascaris* and hookworm only, because *Ascaris* is assumed to be very similar to *Trichuris* in terms of age intensity profiles, excepting drug efficacy against *Trichuris* is known to be lower than for *Ascaris*.

**Figure 6.**
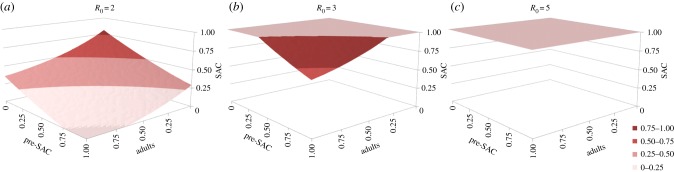
Critical treatment surfaces for *Ascaris* under annual treatment with *R*_0_ = 2, 3, 5 (*a–c*, respectively). Axes represent fraction effectively treated in each age group. Points above the surface lead to elimination within 20 years. Parameters: *z* = 0.94, *k* = 0.5, *σ* = 1 per year, *β* = *ρ* = (1.15, 1.15, 1, 0.52). (Adapted from Truscott *et al*. [40].) (Online version in colour.)

Numerical analyses of the model where deposition is equal to uptake (hence 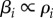 for observed age class, *i*) reveal that treating only SAC will rarely extinguish transmission, except in very low *Ascaris* and *Trichuris* transmission settings [40]. For example, as illustrated in figure 6*a*, when *R*_0_ = 2 only if the SAC age class is treated with an efficacy above 80% is treatment of other age groupings unnecessary to get to the breakpoint. Once *R*_0_ > 2 some of either adults, pre-SAC or both must be treated as well, at high coverage levels (figure 6*b,c*). In most settings where *Ascaris* and/or *Trichuris* are prevalent, *R*_0_ exceeds 2.5 in value (table 1). Treating both SAC and pre-SAC, as is sometimes done in control programmes, with high coverage (greater than 80%) can result in crossing the breakpoint, but only when transmission intensity is low to medium. For the highest transmission setting (figure 6*c*), the model predicts that the contribution of infants alone will prevent chemotherapy eliminating the parasite within a 25 year time frame.

**Figure 7.**
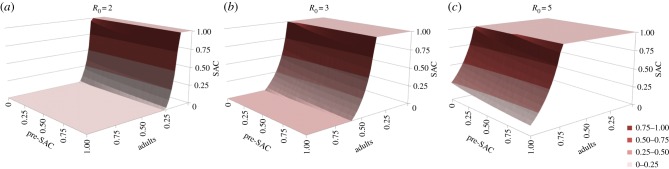
Critical treatment surfaces for hookworm under annual treatment with *R*_0_ = 2, 3, 5 (*a–c*, respectively). Axes represent fraction effectively treated in each age group. Points above the surface lead to elimination within 20 years. Parameters: *z* = 0.92, *k* = 0.4, *σ* = 0.5 per year, *β* = *ρ* = (0.5, 0.5, 1, 2). (Adapted from Truscott *et al*. [40].) (Online version in colour.)

The impact of treating a particular age group is dependent on both the contact that it has with infectious material in the reservoir (as defined by the parameter *β_i_* in equation (2.6)) and on the fraction of the population that it contains (table 2). In the case of hookworm, adults have the highest contact rate and also constitute the largest part of the population. As figure 7 shows, the required treatment levels to achieve elimination are dominated by the coverage of the adult population. The coverage of SAC and particularly pre-SAC has little impact on elimination [40].

In medium and high transmission settings, if either *Ascaris* or *Trichuris* is the most prevalent infection, then the pre-SAC and SAC individuals appear to contribute most to transmission (based on the MCMC estimates of age-related infection rates from age–intensity profiles). In these circumstances, pre-SAC and adults must be treated as well as SAC, with coverage dependent on the value of *R*_0_. For very high values (e.g. *R*_0_ = 5, figure 6*c*), coverage must be above 80% for all treatable age groupings if treatment is annual.

**Figure 8.**
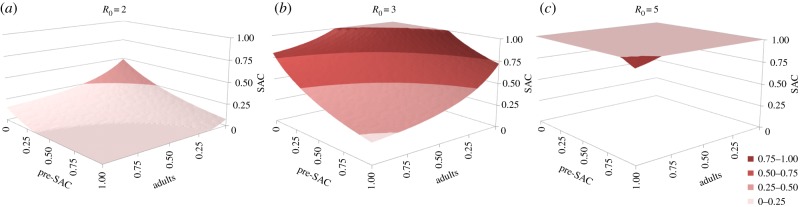
Identical to figure 6, but with treatment of *Ascaris* every six months. (Adapted from Truscott *et al*. [40].) (Online version in colour.)

Current guidelines recommend that treatment is administered every 12 months in low-to-medium transmission settings and every six months in high transmission settings (WHO, 2006, 2011). The model results show that the target coverage level can be reduced if treatment is administered more frequently, such as every six months. This is shown for *Ascaris* in figure 8 where in low-to-medium transmission settings the breakpoint can be attained by treating only SAC (at more than 70% for *R*_0_ = 2 and more than 90% for *R*_0_ = 3). In high settings, treatment levels that do not trigger crossing the breakpoint surface, will have to be sustained indefinitely to avoid a return to pre-control levels. Increasing treatment frequency (every four months) and/or changes in behaviour and sanitation to restrict contamination of the environment with infective stages (and hence lower the value of *R*_0_) will be required if elimination of transmission is to occur.

**Figure 9.**
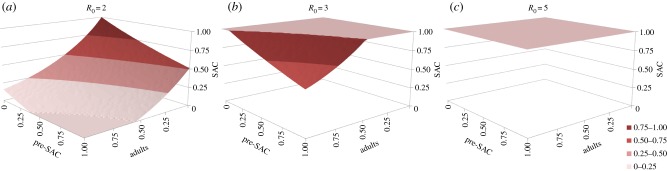
Critical treatment surfaces for *Ascaris* under annual treatment with *R*_0_ = 2, 3, 5 (*a–c*, respectively). As for figure 6, but with egg contribution rate, *ρ*, constant across all age classes. (Online version in colour.)

**Figure 10.**
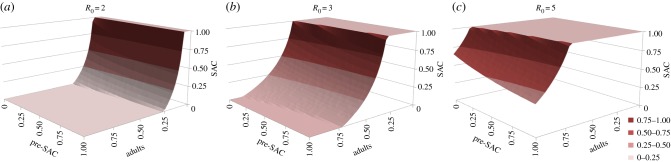
Critical treatment surfaces for hookworm under annual treatment with *R*_0_ = 2, 3, 5 (*a–c*, respectively). As for figure 7, but with egg contribution rate, *ρ*, constant across all age classes. (Online version in colour.)

**Figure 11.**
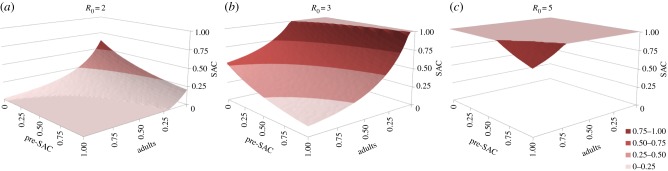
Identical to figure 9, but with treatment of *Ascaris* every six months. (Online version in colour.)

As a sensitivity analysis, we performed these analyses for the scenario in which all age-groups contribute to the infective pool only in proportion to their egg output (*ρ* = 1 for all age groups), as illustrated in figures 9–11. In comparison with our initial assumption, this means that pre-SAC contribute fewer eggs to the reservoir while adults contribute more. In the case of *Ascaris* (figure 9), higher levels of SAC coverage are required for a given adult level, and lower levels for a given pre-SAC level, making the surface ‘twist’ in favour of pre-SAC treatment for effective elimination. For hookworm (figure 10), the threshold coverages are less qualitatively different than before (figure 7). This is due to the continued dominance of adults in transmission. For six-monthly treatment of *Ascaris* (figure 11), the qualitative differences are similar to those for one-yearly treatment. Overall, this comparison demonstrates that understanding who contributes most to infection can be important in designing control programmes and the need for additional epidemiological studies before large-scale roll out of extended treatment guidelines.

**Figure 12.**
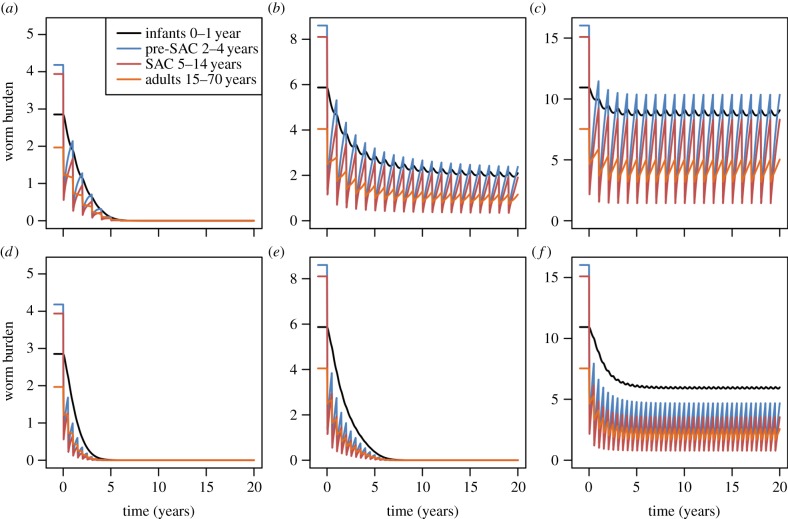
Numerical solutions of the model for *Ascaris* in different transmission and treatment interval settings. Columns represent *R*_0_ = 2, 3, 5, left to right, respectively. Top row represents annual treatment and bottom row six-monthly treatment. Infants (black) are untreated, pre-SAC (blue) and SAC (red) have 90% coverage and adults (orange) have 40% coverage. Other parameter values are as defined in figure 6.

**Figure 13.**
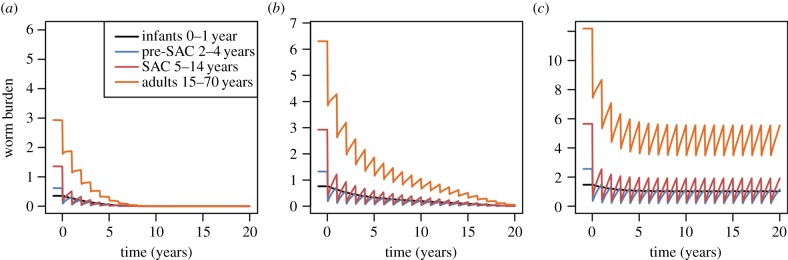
Graphs (*a*), (*b*) and (*c*) are for hookworm, where *R*_0_ is set at 2 for (*a*), 3 for (*b*) and 5 for (*c*). Treatment is annual; treatment coverage is as defined in the legend to figure 9 and parameters as defined in figure 7.

The duration of time over which regular treatment must take place to cross the breakpoint is illustrated in figure 12 (*Ascaris*) and figure 13 (hookworm). For low values of *R*_0_, the breakpoint can be crossed with five years at high (90%) treatment coverage. For medium-to-high transmission settings (*R*_0_ ≥ 3), repeated six-monthly treatment must continue for 10–15 years or longer (at high *R*_0_ values).

**Figure 14.**
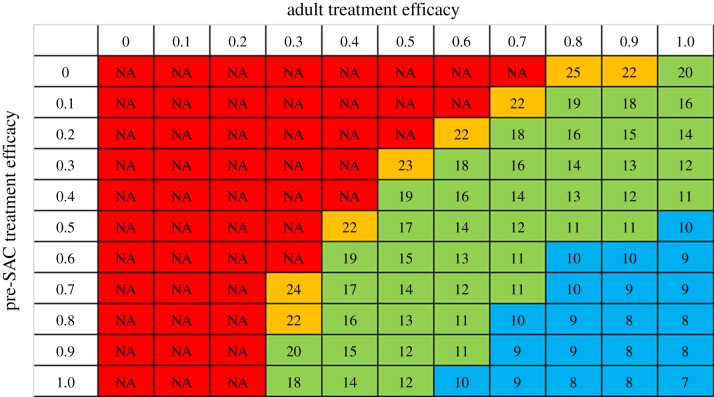
Number of rounds of six-monthly treatment to achieve *Ascaris* elimination as a function of pre-SAC and adult coverage. Coverage of SAC (75%). Table in the figure represents a horizontal slice through figure 11*b*. (Online version in colour.)

We can summarize these dynamics by looking at the number of treatments required to reach the breakpoint. As one would expect, the higher the treatment coverage, the fewer rounds of treatments required to reach the breakpoint. As an example we look at six-monthly treatment of *Ascaris* and coverage among pre-SAC and adults for 75% coverage among SAC (figure 14). For low coverage levels, the programme does not reach the breakpoint within 25 rounds of treatment. As with the coverage surfaces, there is a highly nonlinear relationship between coverage levels and number of rounds required, with the number of rounds being halved to around 10 rounds when coverage is very high. This further illustrates that, even with six-monthly treatment, where the breakpoint can be reached, these programmes may have to be in place for up to a decade to break the transmission cycle.

**Figure 15.**
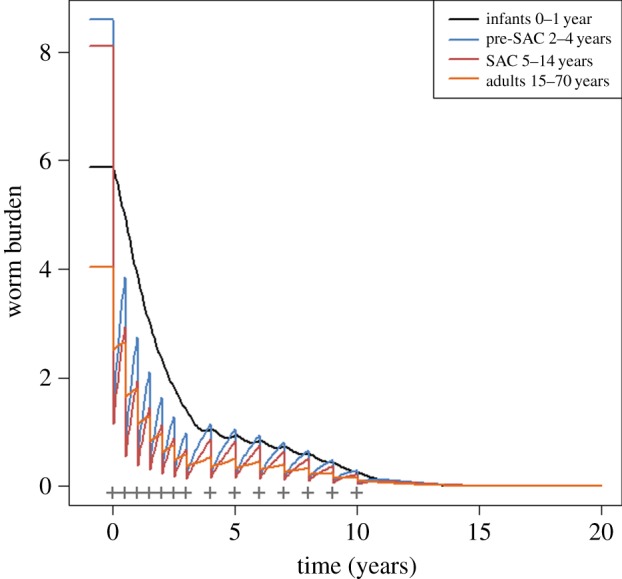
Elimination of *Ascaris* achieved with lengthened treatment interval in final stage (six intervals of six months followed by seven intervals of a year). *R*_0_ = 3; treatment levels and parameters as in figure 9. Treatments represented by grey crosses.

If worm burdens are lowered by repeated treatment, but have not as yet been driven below the breakpoint, then lengthening the treatment interval typically serves only to generate a bounce back to a new equilibrium worm burden above the breakpoint where transmission continues in the community. However, the closer the reduced worm load is to the breakpoint, the longer the bounce back time. As such, treatment intervals can be lengthened once worm loads have been reduced to very low levels (figure 15). A fuller treatment of this time-dependent dynamics is presented in [39].

For all but high transmission settings, community-based chemotherapy alone, covering more than just pre-SAC and SAC, can interrupt the transmission of the common STHs, providing treatment coverage is high, frequent and continued for more than 15 years. Some lengthening of the between treatment intervals can take place once worm loads are reduced to low levels (means less than two per host) after many repeated rounds of treatment.

## 4. Discussion

The aims and objectives of this paper were to examine the optimal intervals between rounds of chemotherapy and coverage levels for different species mixes of STHs, for different age groups treated and for different transmission intensity settings (low, medium and high), to reduce worm loads to very low levels or to cross a breakpoint below which self-sustaining transmission ceases. Analyses were presented on where breakpoints in transmission might lie for the different parasites, again in different transmission intensity settings. An attempt was made to redefine low, medium and high transmission settings and measures of the basic reproductive number *R*_0_, because it is believed that these epidemiological measures are more informative than those based on the prevalence of infection. We used a combination of methods, including data analyses, parameter estimation and numerical analysis based on mathematical models of the transmission dynamics of helminth infections of humans.

The sexual nature of reproduction in human helminth parasites creates a breakpoint in transmission owing to the need for female worms to be fertilized by a male partner to produce viable infective stages [7]. Analyses present in this paper suggest that the breakpoint for STH parasites typically occurs at a very low level (below one parasite) owing to the aggregated distributions of worm numbers per host. As a result, at baseline endemic levels, the impact of sexual reproduction is negligible [7]. However, periodic chemotherapy regularly reduces the worm burden in sections of the population (if only temporarily) to levels at which sexual reproduction becomes a limiting factor. Sexual reproduction dynamics can therefore have a large effect on the parasite in this context. It is shown in figures 6 and 7 that repeated rounds of chemotherapy with high coverage of the whole population can take average burdens below this breakpoint. This situation pertains only in the absence of immigration of infected persons from neighbouring villages and towns who may repopulate the pool of infective stages. The level of coverage of the total population required to achieve this goal is high for moderate to high levels of transmission prior to the start of treatment—often being of the order of 80–90% across all age classes. Somewhat lower levels of treatment coverage will be adequate for low *R*_0_ values (between 1 and 1.5).

Irrespective of the dominant parasite (*Ascaris* or hookworm), beyond the very lowest transmission levels (*R*_0_ = 2), treating SAC alone will never lower average worm burdens near to the breakpoint. Very high levels of coverage (greater than 90%) will suffice if pre-SAC and SAC are treated if *Ascaris* is the dominant STH and *R*_0_ is low, because a large fraction of the total worm population is harboured in these age groupings ([8]; figure 4). This is not the case for hookworm where the majority of the worms are harboured by adults (figure 4). The WHO category to define high levels of infection (prevalence greater than 50%), covers both medium and high levels of transmission. In this case, a treatment programme that effectively covers the adult population is essential to cross the breakpoint in transmission for all the dominant STH species.

The example of hookworm raises a possible issue for policy implementation. The rationale behind school-based intervention is, in part, to alleviate morbidity and improve developmental and educational outcomes for children in the long term. However, reduction in the worm burden and hence morbidity in children may be best served by treating the adult population and indirectly reducing childhood exposure to parasites.

The frequency of treatment required to lower mean worm loads below the breakpoint depends on the life expectancy of the dominant parasite (estimates suggest life expectancies of one year for *Ascaris* and two or more years for *Necator*), and the magnitude of the basic reproductive number *R*_0_. The shorter life expectancy requires more frequent treatment. Every six months is predicted to be necessary for *Ascaris* with moderate to high levels of transmission (*R*_0_ values greater than 2), whereas every year for hookworm provided transmission is not too intense*. Trichuris* is similar to *Ascaris* in terms of life expectancy and hence a six-monthly treatment frequency is ideal.

Comparison of figures 6–8, where egg contribution per individual matches infectious contact, and figures 9–11, where egg contribution is uniform across the population, shows a significant difference. The theoretical basis for the impact of contact rate and individual egg contribution has already been investigated in a simpler model [39]. This feature of the infection process is quite difficult to measure. For example, the endemic age profile of worm burden is not dependent on it. Information can only be recovered from detailed data on parasite bounce back after treatment or by direct behavioural studies within affected communities. Planned longitudinal studies will hopefully lead to better estimates.

The economic evaluation of any programme for helminth elimination will depend in a complex way on the details of drug distribution logistics, but will clearly depend strongly on required levels of effective coverage and overall numbers of treatments necessary. The table in figure 14 shows that there is a trade-off between coverage and number of treatment rounds of a subtle nonlinear nature. It is not immediately clear what would constitute the economically optimal intervention. Future work will use these models as a basis for investigating this area.

In general, a relaxation in the extent or frequency of coverage will lead to a rapid recovery of the parasite population to higher levels. However, as illustrated in figure 15, the effect of sexual reproduction allows for some relaxation in effort while still maintaining control, when burdens have already been driven low. This phenomenon has potential economic consequences for programme design. It suggests that intensive effort in the early stages of control could result in better control in the long term for the same expenditure of resources. To take advantage of such an approach would require close monitoring of community worm burden levels to avoid bounce back in the parasite population.

As repeated rounds of treatment lower the average worm burden, increasing the interval between treatments significantly affects both the likelihood of crossing the breakpoint and the time taken to get to low intensity of infection levels. The numerical studies therefore suggest that a fixed interval as determined by the intensity of transmission prior to the start of treatment should be maintained throughout the 10 year (medium and high transmission sites) or five year (low transmission areas) control programmes. After that, if parasites persist at low intensities, then longer intervals will suffice.

Coverage levels are critical to success, as are the age groups targeted by the control programme. Even for community-wide treatment programmes, high levels of coverage are required to cross the breakpoint. Pre-SAC and SAC treatment programmes combined are not predicted to be effective excepting in very low transmission areas. This conclusion is independent of which helminth is the dominant infection. These results may be of help in planning mass drug administration programmes in setting out guidelines on the targets to be achieved. An important next step is the design of longitudinal epidemiological studies to test the predictions of the models in different communities. These studies are being designed at present.

The analyses point to the need for some additions to the current WHO guidelines for treating STHs by community chemotherapy. The following suggestions are made.

First, pre-treatment assessments and subsequent monitoring should be based on intensity measures not prevalence. As indicated in figure 5, the current threshold criteria of greater than 50% prevalence simply separates low from medium and high transmission intensity areas. The criterion of less than 20% prevalence seems irrelevant.

Second, once treatment is started and calculations are performed to assess treatment frequency based on how the intensity of infection changes by age (to derive the force of infection and the basic reproductive number), this frequency should be maintained for the duration of the programme. The overall duration of treatment should be for a minimum of 10 years and, irrespective of transmission intensity or parasite species mix, the ideal treatment frequency is six months. The treatment interval can be lengthened after control has reduced average worm loads to very low levels.

Third, long-term control (crossing the breakpoint) requires community-wide treatment. Treating only pre-SAC and SAC will not suffice even in areas of low transmission, especially if hookworm is the dominant STH. If resources are limited, treating only pre-SAC and SAC has reported benefits for morbidity control in children and associated longer terms benefits for treated children. But if parasite control and possible elimination is the goal, community-wide treatment is required. In all cases, high coverage levels are required (80–90%). This may be difficult to achieve if school attendance levels are low or if adults are difficult to access. However, the advice offered to country programme managers must stress the need for high coverage, especially in areas of medium-to-high transmission (figure 7).

Fourth and finally, if nothing else alters (i.e. there are no improvements in hygiene and sanitation), then the guidelines must stress that STH populations will bounce back to pre-control levels within a few years if treatment ceases before the breakpoint is crossed. The numerical studies reported in this paper point to these breakpoints for all three of the major STH parasites lying below a mean worm burden of one parasite per host.

The methodological approach adopted in this study can be varied and future publications will address different model structures (e.g. individual-based stochastic formulations) and other helminth species such as the schistosomes and filarial worms. Initial analyses, however, suggest that our general conclusions are robust for STHs to changes in model structure due in part to the somewhat predictable transmission dynamics of helminth parasites (in the terminology of population ecology, they are *k* selected species [41]). The most significant shortcoming is that of the accuracy of parameter estimates, especially those concerning the calculation of the ‘breakpoint in transmission’. With increased activity in the implementation and monitoring of STH control by chemotherapy, a parallel focus on improving our current estimates of key epidemiological parameters via careful monitoring of reinfection by intensity measures post rounds of treatment is highly desirable. Improved standards of measurement will, concomitantly, permit more precision in the design of community-based STH control programmes,

In many poor rural areas of sub-Saharan Africa and South East Asia, the targets outlined on coverage in these analyses across all age classes, will be difficult to achieve in practice—but it should be an ambition for WHO in the coming decade given sustained drug donations from the pharmaceutical industry. The task can be made less daunting by concomitant efforts in hygienic education and the expansion of efforts to provide and maintain safe water and sanitation (WASH programmes). WASH effectively reduces the value of *R*_0_ and hence can move a high transmission setting down to a medium or low one.

In very poor regions, however, the introduction of WASH can be fraught with problems, as can sustaining such introductions, even in school settings. The latter can have limited access to water and little resource to maintain new sanitation facilities to encourage continued child use.

In areas of high transmission intensity, mass chemotherapy in all age groups plus improvements in hygiene and sanitation are essential. Efforts should thus be made on how to cost-effectively treat adults as they will need to be targeted to achieve the interruption of transmission. Perhaps the largest population-wide treatment programme in recent years has been the Lymphatic Filariasis Elimination Programme, whereby all individuals aged one year and above in lymphatic filariasis (LF) endemic areas are treated with albendazole and ivermectin [42]. As these programmes achieve their LF elimination goals, consideration should be made to maintain the infrastructure to control STH infections.
